# An* In Vivo* Study on Intoxicating Effects of* Nerium oleander* Water Based Extract on Multiorgans of Wistar Rat

**DOI:** 10.1155/2018/4739637

**Published:** 2018-04-23

**Authors:** Muddasir Hassan Abbasi, Sana Fatima, Muhammad Babar Khawar, Shah Jahan, Nadeem Sheikh

**Affiliations:** ^1^Cell and Molecular Biology Lab, Department of Zoology, University of the Punjab, Lahore, Pakistan; ^2^Centre for Applied Molecular Biology (CAMB), University of the Punjab, Lahore, Pakistan; ^3^University of Health Sciences, Lahore, Pakistan

## Abstract

This study was aimed to find histological changes in the extrahepatic organs, hepatic iron deposition, and gene expression of some iron regulatory proteins in rats after sterile muscle abscess during the acute intoxication of* Nerium oleander* leaves decoction. 10 ml/kg of the leaves extract was injected intramuscularly in Wistar rats (200–225 g, *n* = 4). Control animals received saline injection of matched volume. Animals were anesthetized and sacrificed after 3, 6, 12, and 24 h after administration of decoction. Lungs, kidney, spleen, and liver were extracted and processed for histopathological examination while portion of liver tissue was proceeded for iron regulatory gene expression quantification. Sections of all studied organs were found with signs of cellular dysfunction with infiltration of variety of leucocytes. In the lungs section at 3 h time point mononuclear cell infiltrates were observed while in alveolar tissue at 24 h time point dilation and even collapse in some of the alveoli were evident. In kidney sections distortion of renal tubules and epithelial cells with shrinkage of glomeruli was noted at all studied time points. In the splenic section of 12 h time point, degeneration, depopulation, and shrinkage of white pulp have been noted. Distension of the red pulp along with activation of splenic follicles was evident after 24 h onset of APR. Significant changes in the expression of acute phase cytokine and iron regulatory genes were noted. IL-6 and Hepc gene expression were strongly upregulated up to 12 h whereby Tf gene expression showed an early upregulation at 3 h time point followed by downregulation on later points while Hjv gene expression showed an overall downregulation at all study time points compared to control. It is concluded that inherent toxins present in the* N. oleander* can induce acute phase response and cause severe histological changes in the organs and marked changes in the regulation of iron regulatory proteins thus cannot be practiced routinely.

## 1. Introduction

Treatment of various human ailments through natural herbs is in practice since centuries. A large group of inherent phytotoxins have been isolated from plants; however still masses of indigenous population rely on these remedies which may results in toxicological emergencies [1].* Nerium oleander (N. oleander) Linn. *(syn.* N. odorum Soland, N. indicum *Mill) (Apocynaceae) is one of such worldwide cultivated plant and has been reported with wide range of different therapeutic effects (Yang et al., 2005; Wang et al., 2008) [2]. All plant parts both green and dried are used in various ailments without knowing their toxicity. Two toxic cardiac glycosides (cardenolides), oleandrin and neriine, have been isolated from all parts of the plant and reported as positive inotropic, negative chronotropic, cross reactive, and very similar to the toxin in foxglove (*Digitalis*) [3, 4].

Damage due to burn or trauma, surgical procedures, advanced malignancies, immunologically and inflammatory disorders, and so on are some of the common stimuli which can induce a complex and defensive biological mechanism of the body called acute phase response (APR). The changes that occurred during APR are believed to guard the host from further detrimental effects during the acute stage of infection and body organs respond differently during this process [5]. It comprised a series of specific physiological reactions, namely, alterations in hematopoietic profile particularly systemic leukocyte mobilization, variety of plasma proteins, and serum levels of glucocorticoids and cytokines (Ceciliani et al., 2002) [6, 7].

Iron is an important and essential part of numerous cellular metabolic activities and its homeostasis is regulated by a large set of cytokines (IL-1*β* TNF-*α*, IFN-*γ*, and IL-6, etc.) and iron-regulatory genes (ferroportin 1 (FPN-1), hemojuvelin (Hjv), hereditary hemochromatosis gene (HFE), divalent metal transporter 1 (DMT1), transferrin (Tf), Tf receptors 1 and 2 (TfR1, TfR2), natural resistance-associated macrophage protein-1 (Nramp-l), ceruloplasmin, and hephaestin (Heph)) while liver mainly manages body iron by making vivid changes in the expression of such genes (Camaschella, 2005; Ganz, 2006).

During inflammation, hepcidin and Hjv genes behave differently and it has been suggested that, in humans, Hjv gene expression could be modulated by inflammation (Krijt et al., 2004). Similar findings of increase in hepcidin production was reported during inflammation and in iron overload conditions (Balogh et al., 2004).

The study was aimed with the objective to induce APR with* N. oleander* leaves decoction and consider the histological changes in the extrahepatic organs, iron deposition in liver, and changes in the gene expression of some iron regulatory proteins of liver in Wistar rat.

## 2. Materials and Methods

### 2.1. Animals

Male Wistar rats (200–225 g) were kept in a well-ventilated hygienic experimental animal house of Department of Zoology, University of the Punjab Lahore-Pakistan, and maintained as described by Abbasi et al. [6].

### 2.2. Experimental Design

*N. oleander leaves* decoction was made according to the method previously described [8], 10 ml/kg of which was administered intramuscularly in both hind limbs using micropuncture needle (0.25 × 6 mm) to the animals at a volume such that it would permit optimal dosage accuracy without contributing much to the total increase in the body fluid. Control animals received saline injection. All the animals from each experimental set (3, 6, 12, and 24 h time points) were anesthetized (i.p.) with ketamine-distilled water mixture of equal ratio (v/v) (50 mg/ml of ketamine) and then sacrificed at their aforementioned time points after extract induction.

### 2.3. Tissue Sampling and Processing

Lungs, kidneys, spleen liver, and muscles from each animal were collected in separate glass vials. All the organs were rinsed with physiological sodium saline and portion was fixed in 10% formalin. Paraffin embedded sections were prepared and processed by standard methods. Hepatic and extra hepatic sections were stained for Prussian blue iron staining and hematoxylin and eosin (H & E), respectively, from Sigma-Aldrich using the protocol provided by the manufacturer followed by microscopic imaging using a trinocular IRMECO-GmbH model 1M-910, 21493 Schwarzenbek/Germany displayed on a computer via a Scope Tek® (scope photo 3.0).

### 2.4. RNA Isolation and Quantitative Real-Time PCR (qPCR)

Total RNA was isolated from liver tissue sample with Trizol reagent according to the manufacturer's instructions. The RNA was then quantified by measuring the absorbance at 260/280 nm. cDNA was generated by reverse transcription of isolated RNA using the RevertAid First Strand cDNA Synthesis Kit (Fermentas). qPCR reactions were set up for each target gene with the following recipe for each reaction: 0.5 *μ*l of each forward and reverse primer (5 mM), SYBR Green master mix Taq polymerase 8 *μ*l, and cDNA sample 1 *μ*l, and final volume was made up to 20 *μ*l with nuclease-free water. 1 *μ*l of H_2_O was used instead of cDNA for the negative control. Amplification was performed at 50°C for 2 min, 95°C for 2 min, and 95°C for 15 sec to 60°C for 30 sec for 45 cycles in a Corbett research Rotor Gene™ 6000 real-time rotary analyzer.  Table 1 showed the list of primers, whose gene was specifically synthesized (Invitrogen). All samples were assayed in duplicate. Curves of amplification were analysed to measure the Ct value in the linear range of the amplification. The results were normalized to the endogenous control (*β*-actin) and fold change expression was calculated using Ct values by Prism Graph Pad 5 software.

### 2.5. Statistical Analysis

The data were analyzed using Prism Graph pad 5 software (San Diego, CA). Statistical significance was calculated by one-way analysis of variance (AONVA) and Dunnett post hoc test. Significance was accepted at *P* < 0.05. Results are shown as mean ± SEM with *n* = 4.

## 3. Results

### 3.1. Histological Findings

The microscopic studies showed varying degree of cellular changes in the tissue integrity from mild to marked following* N. oleander* decoction administration at varying intervals.

### 3.2. Lungs

**Figure 1:**
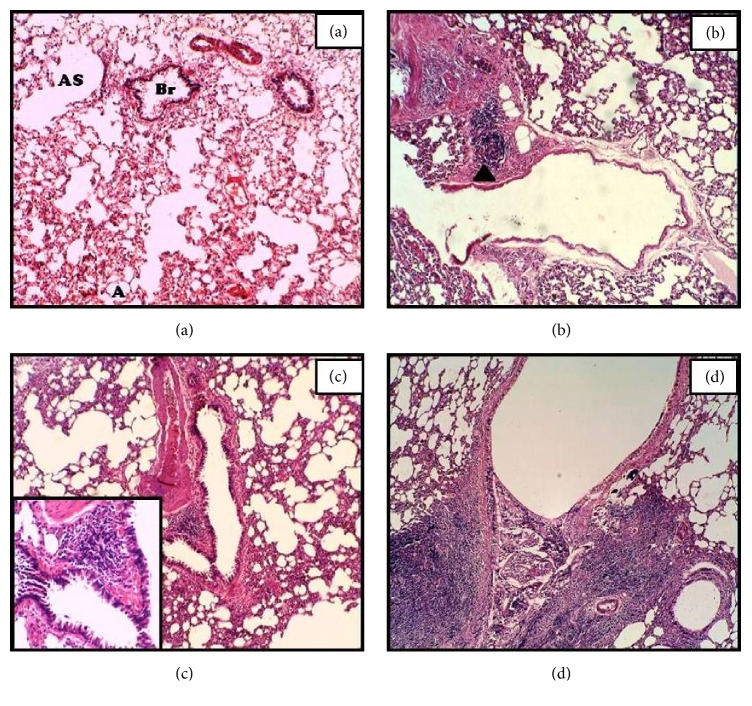
Hematoxylin-eosin staining of lungs sections of* N. oleander* induced APR in rats after 3 (b), 12 (c), and 24 h (d) time course of sterile muscle abscess compared with control (a). The normal architecture of the lungs section showed numerous clear alveoli (A) and alveolar sacs (AS). A bronchus (Br). Cell infiltration was indicated by black arrow head (magnification: = 100x, inset 400x).

Alveoli (A), alveolar sacs (AS), and a bronchus (Br) were observed in section of the control lung tissue (Figure 1(a)). Mononuclear cell infiltrates were observed in tissue section of 3 h and 12 h time point, most frequently around the blood vessel (Figures 1(b) and 1(c)). Alveolar tissue at 24 h time point has shown dilation and even collapse in some alveoli. Massive infiltration along with hemorrhage and extravasation of blood cells and sever negative changes was also observed in this study group (Figure 1(d)).

### 3.3. Kidney

**Figure 2:**
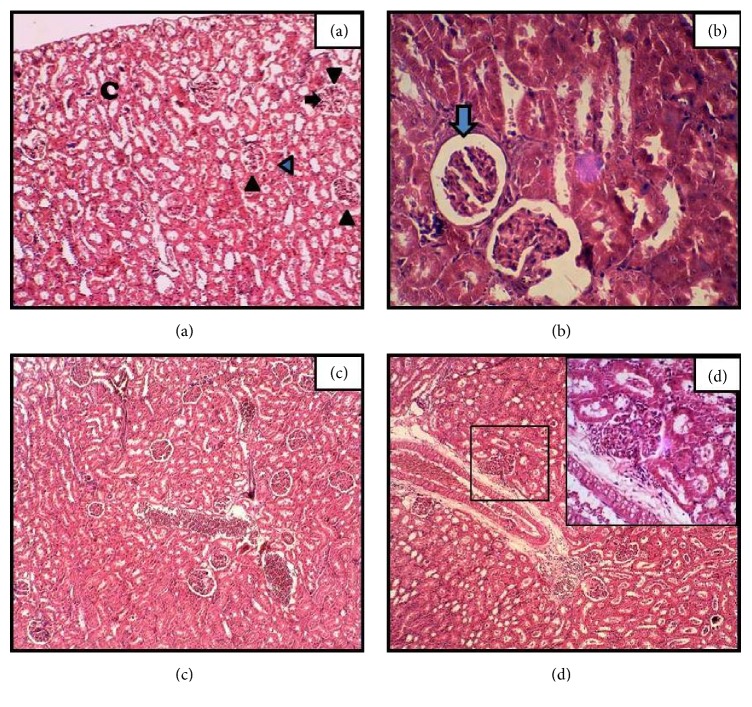
Hematoxylin-eosin staining of kidney sections of* N. oleander* induced APR in rats after 3 (b), 12 (c), and 24 h (d) time course of sterile muscle abscess compared with control (a). Renal corpuscles indicated by black arrow heads, intact glomeruli (black arrow), and proximal tubules (colored arrow head). Treated rats after 3 h showing enlarged bowman's space (colored arrow) (magnification: (a) = 40x; and (c) and (d) = 100x, inset 400x).

Control kidney section showed the renal corpuscles (Malpighian corpuscles) within renal cortex (C). At 3 h time point shrinkage of glomeruli with an enlarged Bowman's space and some cellular desquamation into the lumen was observed (Figure 2(b)). An obvious histological change was noted in renal capsule at 12 and 24 h time point, the sections showing dilatation of capillaries filled with erythrocytes and hemorrhage with enlarged renal capsule with glomeruli. Substantial renal tubular cells were found undergoing necrosis particularly observed 24 h after onset of APR (Figures 2(c) and 2(d)).

### 3.4. Spleen

**Figure 3:**
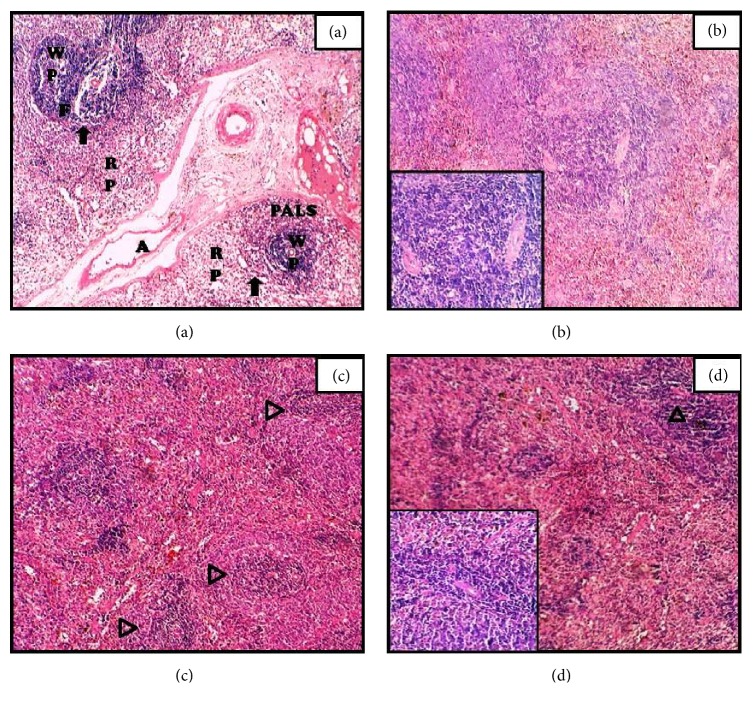
Hematoxylin-eosin staining of spleen sections of* N. oleander* leaves decoction induced APR in rats after 3 (b), 12 (c), and 24 h (d) time course with sterile muscle abscess compared with control (a). Fully developed splenic follicle (F), the periarteriolar lymphoid sheath (PALS), white pulp (WP), red pulp (RP), central artery (A), and the marginal zone shown by black arrow and depopulation is represented with arrow heads. (magnification: 100x; inset 400x).

Morphologically distinct red pulp (RP) and the white pulp (WP) were visible in the control section of spleen. Three subcompartments of white pulp, the periarteriolar lymphoid sheath (PALS), the follicles (F), and the marginal zone, are indicated by black arrow in the tissue section (Figure 3(a)). Cellular disruption and degeneration of the white pulp was seen in tissue sections at 3 h whereas 12 h group showed moderate hyperemic condition with reactive splenic follicles and depopulation (Figures 3(b) and 2(c)). Moderate oedematous, distension of the red pulp along with shrinkage of white pulp, and necrosis of splenic follicles were evident 24 h after the onset of APR (Figure 3(d)).

### 3.5. Liver

**Figure 4:**
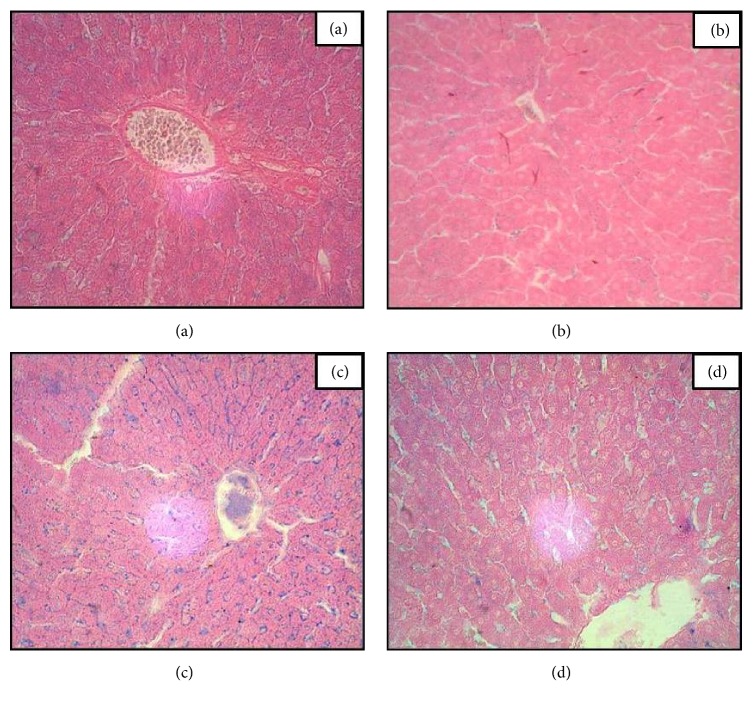
Prussian blue iron staining of hepatic sections of* N. oleander* leaves decoction induced APR in rats after 3 (b), 12 (d), and 24 h (e) time course with sterile muscle abscess compared with control (a). Predominant blue granules were observed in 12 h sections after onset of acute phase reaction while less iron deposits were observed at 24 h time point (magnification = 100x).

Prussian blue iron stained sections after 3 h, 6 h, and 12 h of decoction administration showed extensive iron accumulation while in section of 24 h time point, mild deposition was observed particularly in sinusoidal space. Such distinct bluish granules (ferritin) were predominantly visible within hepatocytes particularly 6 h and 12 h after onset of APR (Figure 4).

### 3.6. Muscle

**Figure 5:**
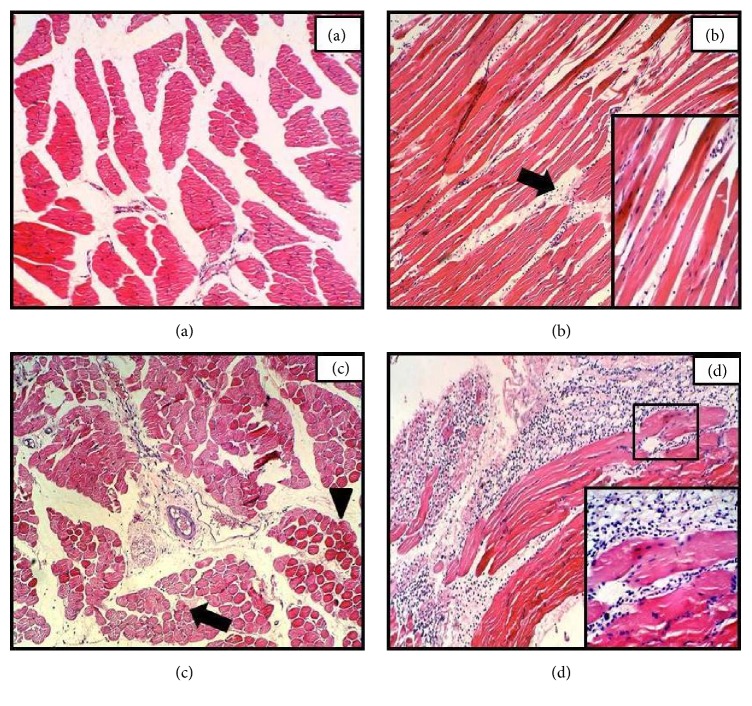
Hematoxylin-eosin staining of muscle sections of* N. oleander* leaves decoction induced APR in rats after 3 (b), 12 (c), and 24 h (d) time course with sterile muscle abscess compared with control (a). Black arrow represents inflammatory cells in the interstitial spaces and among the muscle. Arrow head indicate strong staining in some of the affected individual fibers. (magnification = 100x, inset 400x).

In the 12 h time point section some of the individual fibers were characterized by strong staining of the muscle fibers with loss of cytoplasmic structure and mild infiltration of inflammatory cell compared to control and 3 h sections (Figures 5(a)–5(c)). By 24 h following injection, the histological appearance of the muscles that was markedly changed with necrosis of a single or a few isolated individual muscle fibers was noted. In these muscle fibers, an obvious fragmentation, loss of cytoplasm, and profound infiltration of mononuclear and polymorphonuclear cells were also observed (Figure 5(d)).

### 3.7. Real-Time PCR Analysis

#### 3.7.1. Expression of Acute Phase Cytokine

**Figure 6:**
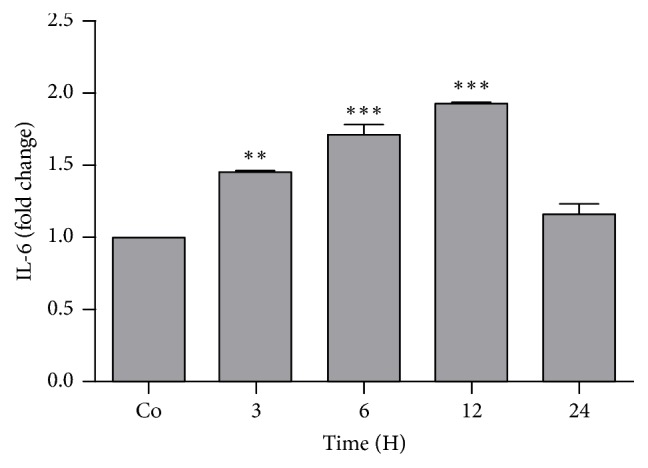
Real-time PCR analysis shown as fold change of mRNA expression of IL-6 in the liver of the rats after administration of* N. oleander*. Significant upregulation in fold change was noted up to 12 h time point. Results were normalized with housekeeping gene ß-actin. Statistical changes are marked with asterisks representing levels of significance (^*∗*^*P* < 0.05, ^*∗∗*^*P* < 0.01, and ^*∗∗∗*^*P* < 0.001; mean ± SEM; *n* = 4).

Real-time PCR analysis revealed gene expression of IL-6 which showed significant upregulation in the liver reaching to the maximum 12 h (1.92 fold ± 0.03) after onset of APR and then downregulation in the expression was noted at later time point (Figure 6).

#### 3.7.2. Expression of Iron Regulatory Genes

**Figure 7:**
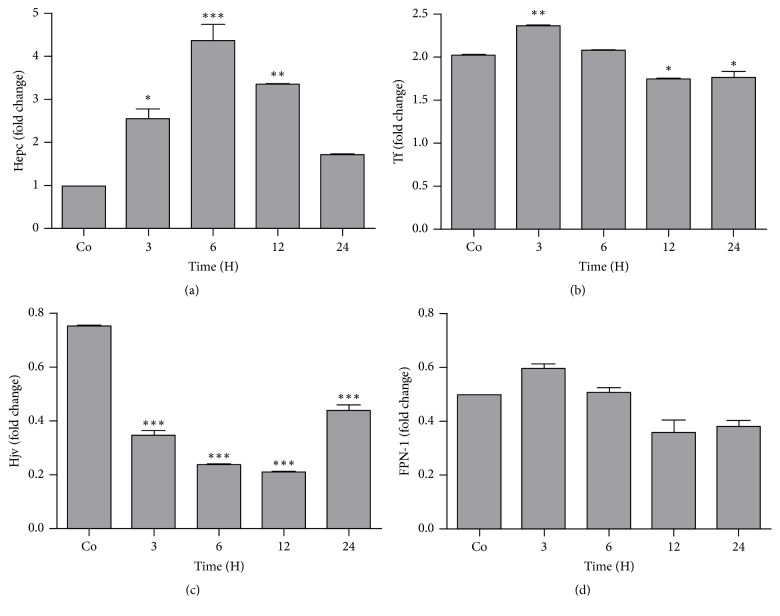
Real-time PCR analysis shown as fold change of mRNA expression of (a) Hepc, (b) Tf, (c) Hjv, and (d) FPN-1 in the liver of the rats after administration of* N. oleander*. Significant upregulation in fold change was noted up to 12 h time point. Results were normalized with housekeeping gene ß-actin. (statistically significant changes are marked with asterisks (^*∗*^*P* < 0.05, ^*∗∗*^*P* < 0.01, and ^*∗∗∗*^*P* < 0.001; mean ± SEM; *n* = 4).

Significant upregulation of Hepc gene expression was noted throughout the course of study compared to control (*P* = 0.0004). Maximum fold change was noted 6 h and 12 h (4.37 ± 0.38  &  3.35 ± 0.01), respectively, after onset of APR (Figure 7(a)). Tf gene expression was significantly upregulated at an earlier time point (3 h) (*P* < 0.01) but later on the gene expression was downregulated compared to control. Hjv gene expression showed downregulation at all-time points with 0.21 ± 0.002 fold decreases at 12 h time point compared to control. A statistically nonsignificant downregulation in FPN-1 gene expression was observed (Figures 7(b)–7(d)).

## 4. Discussion

Herbs are in practice since centuries particularly in Indo-Pak region Hettiarachchi and Kodithuwakku, 1989 [9].* N. oleander* has been reported with wide range of therapeutic properties. Inherent toxins have been isolated from this plant but still masses of indigenous population rely on these remedies which may results in toxicological emergencies [10].

The current study highlights the effects of intoxication of* N. oleander* on some vital organs in rats. Lungs tissue section showed distorted and obliterated air spaces in the current study. This effect might be due to oedema and infiltration of macrophages. The changes and damage observed might be due to an oxidative stress produced by inherent toxicants which can be isolated from all parts of the* N. oleander* and are similar to the toxin in foxglove (*Digitalis*) [3]. These toxins may lead to an imbalance in the production/consumption level of reactive oxygen species (ROS). Abdel-gawad and Atia (2013) noted such derangements in the lungs tissue of DEHP (phthalate plasticizer) treated albino rat group, which is in accordance with the current study. Sheikh and Javed (2009) observed similar tissue changes along with pulmonary edema in swiss albino mice, exposed to 0.5% dilution of cypermethrin in an inhalation chamber. In a similar experimental rat model of intoxication by Abbasi and coworkers [7], 6 h after the respective treatment, destruction of bronchus mucosal folds and alveolar cells with significant nodule-like accumulation of macrophages and mononuclear cells around arteriole was noticed.

Marked degenerative and deleterious histological changes in renal tubules and epithelial cells distortion in general, in the section of kidney, were observed at all study time points. Such changes might be due to the interference of toxins with the structural integrity of the glomeruli and renal tubules which might cause leakage of lysosomal enzymes, thereby causing marked impairment. Adewole et al. (2007) noted similar results in CCl_4_ induced kidney injury in Wistar rats. Brzoska et al. (2003) reported degeneration and hypertrophy of epithelial cells and dilation in the glomeruli in rats exposed to 50 mg Cd/l (as cadmium chloride) and/or 10% (w/v) ethanol (EtOH) for 12 weeks. Our results are also in agreement with previous data in which an oral administration of repeated doses of cypermethrin 5 and 20 mg/kg/day for 30 days displayed hemorrhage and sloughing off renal epithelial cell in the convoluted tubules, shrinkage of glomeruli, and necrosis of renal tubules (Grewal et al., 2010).

Spleen sections revealed shrinkage and cellular disruption of the white pulp at all-time points as compared to the control section. Our results are in agreement with Ciric et al. (2005) and Ebaid and Tag (2012), who observed degenerative and atrophic changes in Monosodium Glutamate (MSG) induced rat spleen. Depopulation of lymphocytes was noted in the white pulp 12 h after onset of APR. This might be due to the immunotoxic effects of xenobiotics or their metabolites in leaves extract as spleen being an important and the largest secondary lymphoid organ to evaluate the treatment-related lesions.

Significant iron deposition in the hepatocytes was evident by Prussian's blue staining; such deposition of iron in the cells is liable for generating ROS, which might lead to cellular and tissue damage. Increase of iron contents in the liver sections at the 6 h time point is an indicative of such condition. Malik et al. [11] and Naz et al. [12] reported similar hepatic iron overload of turpentine-oil induced acute-phase condition.

Sections of muscle obtained after onset of APR revealed signs of damage and cell infiltration. These findings were more profound at 24 h time point in which necrosis of a single or a few isolated individual muscle fibers were more obvious with fragmentation, loss of cytoplasm, and massive infiltration of mononuclear and PMNs cells. The results might be due to the muscle loading or acute injury resulting in inflammation which caused rapid invasion of inflammatory cell population evident in tissue sections. Muscle fiber degeneration might also be due to disturbance in Ca+ ion transporting system of muscle fibers which eventually initiate fiber degeneration. Hassan et al. [13] reported variation in size, splitting, and focal degeneration of myofibers of the gastrocnemius muscle as well as mononuclear cellular infiltration in simvastatin induced male rats which coincide with our findings. Devkota et al. [14] observed hyperplasia of mesenchymal connective tissue in rat skeletal muscle similar to that observed 12 h after the onset of APR in the current study.

Intramuscular injection of leaves decoction induced significant changes in the expression of acute phase cytokine and iron regulatory genes. IL-6 and of hepcidin (Hepc) gene expression were strongly upregulated up to 12 h after onset of APR whereby the maximum level of Hepc gene expression was noted at 6 h time point. The increase in IL-6 gene expression is of significant importance as IL-6 is an important acute phase mediating cytokine. Similar upregulation in the expression of IL-6 was reported in LPS induced model of inflammation (Lee and Beutler, 2009). The components of* N. oleander *decoction might have induced the IL-6 gene expression with subsequent upregulation of hepcidin to regulate serum iron level. Our results are in accordance with the previous findings in which upregulation of several chemokines and acute phase mediators (IL-1*β*, IL-6, and tumor necrosis factor-*α*) has been reported during APR [5, 15]. An early upregulation at 3 h time point followed by downregulation on later points was noted for Tf gene expression while Hjv gene expression showed an overall downregulation at all study time points compared to control. Hepatic FPN-1 gene expression was downregulated after 3 h of injection up to studied time points compared to control. FPN-1 is an inflammatory negative acute phase protein which is concurrent in the study and it provides an inverse relationship with hepcidin. The changes in gene expression showed an inverse relationship between the two genes (Nemeth et al., 2004). Since hemojuvelin (HJV) is the key regulator of hepcidin, a master iron-regulatory peptide. In certain conditions like nonalcoholic fatty liver disease (NAFLD) the expression of HJV has been reported to be downregulated with higher hepcidin levels [16]. This might be due to physiological response to iron accumulation in the liver which is evident in the current study. A similar situation of HJV and FPN-1 genes regulation has been reported by Sheikh et al. [17]. Many feedback mechanisms might therefore be participating in HJV and FPN-1 gene regulation during inflammatory conditions with iron overload.

## 5. Conclusion

Concluding from the results obtained that the excessive use of ethnomedicines including application of* N. oleander *may lead to changes in normal architecture of important organs and cause disturbance in the iron regulatory as well as acute phase cytokine expression thus cannot be practiced routinely.

## Figures and Tables

**Table 1:** List of forward and reverse primers used for real time PCR.

Primers	Forward 5′→3′
	Reverse 3′*←*5′
ß-actin	TGT CAC CAA CTG GGA CGA TA
	AAC ACA GCC TGG ATG GCT AC
Tf	GGC ATC AGA CTC CAG CAT CA
	GCA GGC CCA TAG GGA TGT T
*Hepc*	GAA GGC AAG ATG GCA CTA AGC A
	TCT CGT CTG TTG CCG GAG ATA G
Hjv	ATG CCG TGT CCA AGG AGC TT
	TCC ACC TCA GCC TGG TAG AC
FPN-1	TTC CGC ACT TTT CGA GAT GG
	TAC AGT CGA AGC CCA GGA CTG T
IL-6	GTC AAC TCC ATC TGC CCT TCA G
	GGC AGT GGC TGT CAA CAA CAT
